# Application of transcranial Doppler in cerebrovascular diseases

**DOI:** 10.3389/fnagi.2022.1035086

**Published:** 2022-11-08

**Authors:** Yuxiao Wan, Xiufei Teng, Shiyi Li, Yanchao Yang

**Affiliations:** Department of Anesthesiology, Shengjing Hospital of China Medical University, Shenyang, China

**Keywords:** transcranial Doppler, cerebrovascular disease, clinical application, ischemic stroke, monitoring cerebral vascular occlusion

## Abstract

Transcranial Doppler (TCD) is a rapid and non-invasive diagnostic technique that can provide real-time measurements of the relative changes in cerebral blood velocity (CBV). Therefore, TCD is a useful tool in the diagnosis and treatment of clinical cerebrovascular diseases (CVDs). In this review, the basic principles of TCD and its application in CVD were outlined. Specifically, TCD could be applied to evaluate occlusive CVD, assess collateral circulation in patients with ischemic stroke, and monitor cerebral vascular occlusion before and after thrombolysis as well as cerebral vasospasm (VSP) and microembolization signals after aneurysmal subarachnoid hemorrhage (SAH). Moreover, TCD could predict short-term stroke and transient cerebral ischemia in patients with anterior circulation occlusion treated with endovascular therapy and in patients with anterior circulation vascular occlusion. Additionally, TCD not only could monitor blood velocity signals during carotid endarterectomy (CEA) or carotid artery stenting (CAS) but also allowed earlier intervention through early recognition of sickle cell disease (SCD). Presently, TCD is a useful prognostic tool to guide the treatment of CVD. On the one hand, TCD is more commonly applied in clinical research, and on the other hand, TCD has an increasing role in the management of patients. Collectively, we review the principles and clinical application of TCD and propose some new research applications for TCD.

## Introduction

Transcranial Doppler (TCD), a diagnostic method first proposed by Aaslid et al. (1982), can non-invasively and inexpensively examine the characteristics of blood velocity in the cerebral basilar artery and cerebrovascular hemodynamics. Currently, TCD is increasingly used in the evaluation of cerebrovascular disease (CVD) because it allows continuous measurements, real-time responses, and bedside operation (Lau et al., 2020). TCD is a non-invasive, portable, and radiation-free way to assess cerebral circulation as well as the diagnosis and follow-up of intracranial mass occupying lesions (Blanco and Abdo-Cuza, 2018). CVD mainly refers to abnormal function of the central nervous system induced by stenosis, blockage, or rupture and bleeding of intracranial blood vessels due to various causes. Currently, the incidence of CVD is increasing worldwide (Wang, 2011). A timely and effective response to the changes in cerebral blood velocity (CBV) is important for early diagnosis, formulation of treatment plans, and avoidance of adverse events in CVD patients. At present, digital subtraction angiography (DSA) serves as the gold standard for diagnosing CVD due to its ability to obtain clear vascular images through the process of subtraction, enhancement, and re-imaging of X-ray images. However, the application of DSA is limited in some CVDs because DSA is an invasive test (D’Andrea et al., 2016). Magnetic resonance imaging (MRI) can display intracranial tissue clearly but cannot provide hemodynamic data (Zhou et al., 2021). Therefore, TCD has become an important imaging modality for evaluating CVD.

## Basic principles and development of transcranial Doppler

The development of TCD is based on the Doppler effect. Specifically, low-frequency ultrasound waves emitted by a Doppler probe were transmitted through the skull and reflected by red blood cells that moved within the blood vessels. The frequency difference between the transmitted and reflected waves, known as the “Doppler shift,” is proportional to the moving speed of red blood cells in blood vessels (Robba et al., 2019). Because CBV in the vasculature is laminar, different Doppler frequency shifts can be displayed and form a spectrum of the velocity distribution of individual red blood cells on the TCD monitor. The formula describing the relationship between CBV and Doppler shift frequency was shown as follows:


CBV⁢(cm/s)=(Dopplershift×wavepropagationspeedinthemedium)/(2×emission⁢frequency×cos⁢Θ).

The propagation speed of the wave in the medium is constant. Theta (Θ) is the angle of the emitted wave relative to the direction of blood velocity. If the angle is 0° or 180°, the cosΘ is 1, indicating extremely accurate measurement. Therefore, the angle needs to keep a minimum value during the process of measurement to reduce errors (Purkayastha and Sorond, 2012).

In intracranial Doppler studies, the high-frequency ultrasound cannot effectively penetrate the skull due to weak penetration. Hence, low-frequency ultrasound (2.0–3.5 MHz) is commonly applied in TCD examinations. Moreover, the transmission of sound waves needs to be selected to penetrate the thinner areas of the skull (acoustic windows) in order to cause resonance in the cerebral arteries. There are four major acoustic windows, containing the temporal lobe window, the submandibular window, the orbital hilum, and the suboccipital window (D’Andrea et al., 2016; Couture et al., 2017).

Based on the above principles, the first TCD was established by Aaslid et al. (1982) using low-frequency ultrasound and an appropriate acoustic window, which was a significant breakthrough in imaging technique. However, TCD examinations are dependent on the expertise and experience of operators due to the absence of guidance with two-dimensional imaging, which leads to blindness, randomness, and poor repeatability of results (Hakimi et al., 2020). Therefore, transcranial color Doppler ultrasonography (TCCD) has been developed. TCCD can identify the artery at an anatomical location through combination with the cross-section of the resonance region. In addition, by correcting the angle of the emitted wave and the direction of blood velocity, measurement errors of TCCD can be minimized (Swiat et al., 2009). In summary, TCCD promotes the widespread use of TCD.

## Clinical application of transcranial Doppler

### Evaluation of cerebral artery stenosis/occlusive disease

Intracranial artery stenosis/occlusion mainly caused by intracranial atherosclerosis is an important factor causing ischemic brain disease and an independent factor inducing stroke recurrence (Holmstedt et al., 2013). Currently, TCCD, as the most widely applied method to detect cerebral artery, can examine the degree of stenosis/occlusion of the proximal middle cerebral artery (MCA), anterior cerebral artery, posterior cerebral artery, basilar artery, and bilateral vertebral arteries (VA) by non-invasive, continuous monitoring of the CBV (Finnsdottir et al., 2020; Spence, 2020; Adıyaman et al., 2021). Additionally, compared with the anterior circulation, there is greater tortuosity and variability in the posterior circulation; predictably, the sensitivity and specificity of TCCD are higher in the anterior circulation (Jaiswal et al., 2019). A single-center retrospective study analyzed the stenosis of 720 cerebral arteries from 80 patients by TCCD and magnetic resonance angiography (MRA) (Mowla et al., 2020). The results exhibited higher specificity and accuracy of TCCD than cranial MRA, confirming the suitability of TCCD for the screening of intracranial stenosis.

### Evaluation of compensatory opening of cerebral collateral circulation

Normally, the collateral circulation is in a low-flow state. When there is a pressure difference between two arteries, collateral arteries are recruited to divert blood around obstructions. This recruitment of collateral vessels indicates the presence of diseased blood vessels and the possibility of ischemic stroke (Luo et al., 2017; Saqqur et al., 2018). Evidence has revealed that the collateral grades significantly correlate with infarct size and prognosis in patients with ischemic stroke (Seyman et al., 2016).

The anterior and posterior communicating arteries, as the main pathways of intracranial collateral circulation, can supply continuous perfusion of blood to the brain in the case of cerebral arterial stenosis or occlusion (Guan et al., 2013). Despite acting as the gold standard to evaluate the lesions of collateral circulation, the application of DSA is limited by its adverse effects (cerebral arterial spasm after angiography) and invasive disadvantage. Therefore, non-invasive TCD with a high degree of repeatability is applied in the indirect assessment of collateral circulation. TCD allows for real-time monitoring of the known direction and CBV in intracranial collateral circulation, as well as identification of the direction of these collaterals and the donor and recipient arterial systems (Wei et al., 2019). The Chinese consensus group on collateral circulation for ischemic stroke (Liu et al., 2014) noted that cerebral collateral circulation may increase the blood perfusion at the infarcted site, enhance ischemic tolerance, and improve drug delivery, thereby enhancing therapeutic effects. Hence, continuously monitoring the cerebral collateral circulation by TCD is of great significance to determine the treatment plan and predict the cerebral infarction volume and prognosis of patients with ischemic stroke.

## Guidance on thrombolysis

As for the thrombolytic therapy of patients with acute stroke, TCCD is primarily used to evaluate the degree of cerebral artery occlusion before or during the treatment of intravenous thrombolysis (Mazya et al., 2018). Studies have stated that continuous monitoring of TCCD combined with anticoagulant drugs can greatly increase the success rate of thrombolysis but not increase the incidence of complications such as cerebral hemorrhage (Kramer et al., 2011). A study (Brunser et al., 2016) showed that TCCD examination in the first 4.5 h acute ischemic stroke allowed the treatment plan adjustment by providing more information on CBV, thereby changing the results for some patients.

### Monitoring of subarachnoid hemorrhage and cerebral vasospasm

Cerebral vasospasm (VSP) is a common complication in patients with aneurysmal subarachnoid hemorrhage (SAH), and more than half of these patients suffer neurocognitive decline. There is a direct correlation between the severity of VSP after SAH and the blood velocity in cerebral arteries. TCD contributes to monitoring the angiography of VSP after SAH to guide the timing of diagnostic and therapeutic angiographic interventions; specifically, the CBV in the MCA and VA was measured to obtain a more accurate detection result of VSP (Wang et al., 2018; Santos-Teles et al., 2019; Roa et al., 2020).

In a follow-up study of 47 patients with SAH (Djelilovic-Vranic et al., 2017), the mean CBV was 130 cm/s in the MCA; the sensitivity and specificity of TCD for the detecting of VSP were 73 and 100%, respectively. Furthermore, under the conditions of aneurysm, hypertension, smoking, etc., about a quarter of patients with SAH showed a slight increase of CBV in the first few days by TCD monitoring; at the second week, all patients presented a significant increase in CBV; until the third week, there were about 1/4 of the patients with a slight increase in CBV. The above findings indicate that an ischemic condition may be present in the brain. TCD is the preferred method for managing VSP after SAH because it allows a long-term continuous monitoring and can prevent delayed cerebral ischemia.

### Monitoring of microemboli

Microemboli (MES), primarily composed of gas and debris generated during extracorporeal circulation and surgery, are generally less than 500 μm in diameter. Previously, MES could not be detected. Now, TCD provides real-time monitoring of MES in cerebral circulation based on the backscatter of ultrasound waves from emboli. Specifically, the backscatter of ultrasound from gaseous emboli is higher than that from the solid emboli and red blood cells (Wojczal et al., 2002). Studies have demonstrated that patients with MES are prone to re-occurrence of cerebral ischemia (HR: 4.90, 95% CI 2.16–11.09, *P* < 0.001) and a more severe functional impairment after thrombosis (OR: 3.31, 95% CI 1.22–8.99, *P* = 0.019) (Das et al., 2020). Sheriff et al. (2020) utilized TCD to monitor microembolic signals in the patients after successful thrombectomy and the patients with anterior circulation occlusion. In brief, MES signals were detected in 43 of 111 patients, with a median rate of 4 times/h; on 24-h computed tomography, similar infarct volume [adjusted β = 11.2 (95% CI: −46.6 to 22.9), *P* = 0.51] was observed in patients with or without MES; besides, MES also predicted new embolic events [Cox hazard ratio 6.78 (CI 95% 1.63–27.8), *P* = 0.01]. The above outcomes verified that TCD had a good predictive value for short-term stroke and transient cerebral ischemia by monitoring for MES signals in the patients with anterior circulation occlusion treated with endovascular therapy and anterior circulation vascular occlusion.

### Carotid endarterectomy and carotid artery stenting monitoring

Carotid endarterectomy (CEA) and carotid artery stenting (CAS) are important procedures to reduce carotid artery stenosis, improve blood velocity, and prevent ischemic stroke. However, intraoperative cerebral ischemia and postoperative hyperperfusion-induced cerebral hemorrhage bring a high mortality to patients receiving CEA or CAS treatment (Udesh et al., 2017; Moniche et al., 2020). Therefore, it is necessary to provide a real-time monitoring for the CBV during the operation. By virtue of TCD, clinicians can take appropriate measures to restore intracranial blood velocity according to the changes of CBV, thereby avoiding intraoperative cerebral ischemia and postoperative cerebral hemorrhage. In a retrospective study (Spence, 2017), 24 patients with cerebrovascular complications were screened using TCD to monitor various stages of CEA in 500 patients with CEA. Clinicians took the corresponding measures in light of TCD signals, and patients with permanent postoperative defects decreased from 7% of the first 100 cases to 2% of the last 400 cases (*P* < 0.01). The study of CAS (Garami et al., 2009) also stated that intraoperative cerebrovascular complications and postoperative neurological damage could be effectively reduced after the application of TCD in the submandibular window and the transtemporal window to monitor each stage of CAS in real time. The use of TCD in patients undergoing CEA contributes to the early detection of intraoperative and postoperative cerebral hyperperfusion, which is the key to reducing postoperative cerebral hemorrhage (Tong et al., 2014). In a nutshell, the surgical outcome and prognosis could be improved by the intraoperative and postoperative monitoring with TCD in patients undergoing CEA and CAS.

### Prediction of sickle cell disease

As a genetic disorder, sickle cell disease (SCD) is characterized by chronic hemolysis-caused chronic anemia. Inflammation and intracranial arterial stenosis can be induced by the interaction of sickled red blood cells with endothelial cells, which is highly associated with stroke in children. By the way, the transfusions of blood transfusions can reduce the probability of stroke. TCD has been confirmed to accurately predict the risk of stroke in the children with SCD. Therefore, TCD can be used to continuously monitor the children with SCD and allows for earlier recognition and preventive treatment of SCD (Crow, 2020; DeBaun et al., 2020). In the previous study (Kirkham and Lagunju, 2021), TCD screening showed that the incidence of stroke was reduced by a factor of 10 in children (with an average maximum velocity >200 cm/s) receiving prophylactic chronic blood transfusion. Similar findings were found in another stroke prevention trial (Fullerton et al., 2004). Briefly speaking, TCD was used for long-term monitoring of children (with SCD but without a history of stroke) who had a threshold of 200 cm/s; prophylactic blood transfusion was given based on the average flow velocity observed during monitoring; after that, the risk of stroke was reduced by 92% in children.

### Weaknesses of transcranial Doppler

Transcranial Doppler is highly dependent on the operator’s experience and affected by various pathological and physiological parameters, so the technicians are required to have the detailed three-dimensional knowledge of cerebral vascular anatomy. Additionally, the results of TCD are affected by a poor acoustic window due to individual differences, including the skull thickness around the acoustic window or other reasons causing attenuation of ultrasound energy.

Various physiological parameters (age, gender, blood viscosity, and blood pressure) also affect the measurement of CBV using TCD. It is reported that CBV was decreased at a rate of about 0.3–0.5% per year with the growth of age. Moreover, age is regarded as one of the most influential factors in the blood velocity of MCA. Generally, the CBV in young women is higher than that in young men (Lefferts et al., 2020; Alwatban et al., 2021). Besides, hematocrit and viscosity are negatively correlated with CBV, while blood pressure is positively associated with CBV (Santos-Galduroz et al., 2012; Willie et al., 2014). Furthermore, factors such as mental state and temperature have also been reported to affect CBV. Therefore, the evaluation of CVD based on CBV still needs additional data support.

## Future outlook

### Cerebral autoregulation

Cerebral autoregulation is an intrinsic property of blood vessels and maintains a relatively constant CBV by rapidly adjusting cerebrovascular resistance and compensating for fluctuations in cerebral perfusion pressure (Tan and Taylor, 2014). Abnormal cerebral autoregulation occurs in many diseases, such as stroke, SAH, and eclampsia (Castro et al., 2018; Kamerić et al., 2021). Currently, studies of CBV regulation rely on steady-state blood pressure. This method is time-consuming and requires invasive procedures, such as the Kety-Schmidt technique using Xenon Xe 133 as a tracer. Dynamic vascular changes cannot be identified with the technique relying on steady-state blood pressure due to a lack of temporal resolution. As a powerful and non-invasive tool with a high temporal resolution, TCD ultrasound is useful for assessing dynamic changes of CBV under various conditions, including changes in arterial pressure. TCD has become the most commonly used tool in the study of CBV regulation in humans. In clinical practice, the change of CBV was measured by TCD after the alteration of blood pressure to evaluate cerebral autoregulation. Rynkowski et al. (2019) evaluated the cerebral autoregulation in SAH patients by TCD to predict postoperative functional recovery and prognosis. Shortly speaking, the blood pressure was changed by temporarily compressing the common carotid artery, and the change of CBV during this period was measured by TCD; cerebral autoregulation was considered to be preserved when an increase ≥9% of baseline systolic velocity was present. The results showed that the injury of cerebral autoregulation was associated with poor prognosis of patients with SAH. There are many ways to change the blood pressure of a patient in clinical practice, such as changing posture, drug intervention, and Valsalva maneuver (Mankovsky et al., 2003; Vagli et al., 2020). Although TCD cannot directly measure the self-regulation of brain parenchyma, the change of CBV in main arteries is closely related to cerebral perfusion. What is more, TCD also provides a simple method for the clinical evaluation of cerebral autoregulation.

### Default mode network

Default mode network (DMN) is a hot spot in the research on resting-state brain functional networks; despite no studies on this subject previously, a large amount of data have been accumulated. In 1990, Raichle used fMRI to track the changes in blood oxygen in the human brain during non-directed mental activity. In his finding, when the human brain was awake and in a resting state, strong signals as well as strong and regular activities were presented in certain areas of the brain. According to this finding, Raichle and Snyder (2007) proposed the hypothesis of resting-state activity.

Buckner (2013) defined the DMN of the brain in a meta-analysis of brain imaging. In their meta-analysis, the DMN was an anatomically defined brain system. Furthermore, functional anatomy suggested that the medial temporal lobe system provided the information from previous experiences in the form of memory and association, and the medial prefrontal lobe system contributed to the construction of self-relevant psychology. Also, the importance of DMN in the understanding of mental disorders was discussed (autism, schizophrenia, Alzheimer’s disease, etc.). Alterations in DMN have been reported in patients with Alzheimer’s disease (Li et al., 2012), schizophrenia (Orliac et al., 2013), and brain injury (Abbas et al., 2015) even before the onset of clinical symptoms. In fact, patients with normal TCD and MRI/MRA still have cognitive deficits dominated by attention, memory, and executive functions that do not adequately explain poor cognitive performance (Bernaudin et al., 2000; Brousse et al., 2015). In a study of SCD (Colombatti et al., 2016), the DMN was assessed by TCD, MRI, MRA, etc. The outcomes revealed that a selective disruption of connections between related brain regions in children with SCD may lead to cognitive decline and altered functional brain dynamics. Therefore, the combination of functional MRI, TCD, and other brain imaging techniques in the study of brain anatomy and functional alterations in DMN should help to evaluate the cognitive dysfunction of patients.

## Conclusion

Transcranial Doppler is an inexpensive, simple, and non-invasive imaging method that is widely used in clinical practice. In brief, TCD can aid in the clinical diagnosis, intraoperative monitoring and management, and prognosis evaluation of CVD by effectively reflecting cerebral hemodynamic characteristics. Besides, TCD plays a vital role in the diagnosis of ischemic stroke, VSP, and cerebral MES, the intraoperative management of CEA and CAS, the evaluation of collateral compensation, and the monitoring of children with SCD and other CVDs. Notably, TCD also plays an indispensable role in the research of cerebrovascular physiology including cerebral autoregulation.

## Author contributions

YW and YY designed the study and wrote the manuscript. XT and SL collected and analyzed the data. All authors contributed to the article and approved the submitted version.
